# Oral Anticoagulant Therapy of Patients With Atrial Fibrillation in Cardiology, Internal Medicine, and Surgery: Temporal Trend

**DOI:** 10.1002/mco2.70487

**Published:** 2025-11-16

**Authors:** Mingjie Lin, He Huang, Juntao Wang, Hui Sun, Xingsheng Xu, Yan Zhang, Wenqiang Han, Min Chen, Kui Dong, Yingcui Wang, Beian You, Guihua Yao, Jingquan Zhong, Congxin Huang

**Affiliations:** ^1^ Department of Cardiology Cheeloo College of Medicine Qilu Hospital (Qingdao) Shandong University Qingdao China; ^2^ State Key Laboratory for Innovation and Transformation of Luobing Theory Key Laboratory of Cardiovascular Remodeling and Function Research Department of Cardiology Chinese Ministry of Education Chinese National Health Commission and Chinese Academy of Medical Sciences Qilu Hospital of Shandong University Jinan China; ^3^ Department of Cardiology Renmin Hospital of Wuhan University Wuhan China; ^4^ Shinall Technology Wuhan China

**Keywords:** atrial fibrillation, cardiology, China, oral anticoagulants, stroke prevention

## Abstract

This study aimed to assess the status of oral anticoagulant (OAC) therapy among Chinese patients with atrial fibrillation (AF) across various hospital departments, influenced by recent changes in medical insurance policies. It retrospectively analyzed data from 70,187 AF patients treated between January 2018 and December 2023 across 20 hospitals. The average patient age was 72.3 years, with 54.4% male. The study found a significant increase in OAC use over time, particularly in cardiology, where the usage rose from 29.8% pre‐2018 to 68.8% in 2021–2023. However, OAC usage in non‐cardiology departments remained below 50% during the same period. Tertiary hospitals had higher OAC prescription rates compared to non‐tertiary hospitals. Despite 77.4% of the cohort being at high risk for stroke, their OAC usage rates were not higher than the non‐high‐risk group. Factors such as advanced age, history of bleeding, hemoglobin levels, and concurrent antiplatelet therapy hindered OAC use, while upstream treatments facilitated its acceptance. The study concludes that while progress has been made in OAC prescription in China, significant gaps remain, especially in internal medicine and surgery departments, necessitating targeted interventions and better interdisciplinary collaboration for improved patient outcomes.

## Graphical abstract

The multicenter retrospective study reviewed electronic medical records of 70,187 AF patients from 20 hospitals between January 2015 and December 2023. The main findings were that OAC usage was significant increase particularly in cardiology (29.8% pre‐2018 to 68.8% in 2021–2023), while still below 50% in non‐cardiology departments; influent factors for OAC prescription were varied among departments. Significant gaps of OAC remain in internal medicine and surgery departments.

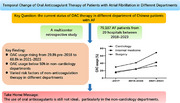

## 1 Background

Atrial fibrillation (AF) is a prevalent cardiac arrhythmia that poses significant healthcare challenges worldwide. Its prevalence is continually rising, contributing to increased incidence rates and substantially elevating the risk of mortality and morbidity, particularly through complications such as ischemic strokes [1, 2, 3, 4]. Globally, AF affects between 2% and 4% of the adult population, highlighting its widespread nature [1]. Surveys recently conducted in Chinese communities indicate that around 1.8% of adults over 45, nearly 8 million individuals, suffer from AF [5]. Despite progress in the treatment protocols for AF, stroke prevention remains a pivotal aspect of patient management—an area necessitating a vigilant approach given the profound impact strokes have on patient outcomes [1, 2].

One of the primary strategies in stroke prevention for AF patients is the administration of oral anticoagulants (OACs). These include traditional vitamin K antagonists (VKAs, such as warfarin) and newer (non‐VKA oral anticoagulants [NOACs], including dabigatran, rivaroxaban, apixaban, and edoxaban), which have been shown to reduce stroke risk by approximately 66% [1, 2]. Despite their proven efficacy, the utilization of OACs on both a global and local scale remains suboptimal [6, 7]. This is particularly pronounced in China, where the anticoagulation treatment rates among AF patients range from a mere 10% to 50% [8, 9]. These figures are significantly lower compared to European, American, and Japanese populations, as illustrated by the findings of the Global Registry on Long‐Term Oral Antithrombotic Treatment in Patients with Atrial Fibrillation (GLORIA‐AF) study [7]. According to this study, during phase II (2013‒2014), only about 20% of patients at high risk of stroke were receiving OACs in China [6]. Although this improved to about 40% by phase III (2015‒2016), it still lagged behind Japan, where 80% of similar patients received these treatments during the same period [6, 10]. Additionally, among a registered nationwide cohort of 137,181 hospitalized AF patients surveyed across 362 tertiary medical centers between November 2017 and October 2018, a high OAC prescription rate of 79.1% was reported [11]. Contrastingly, the latest data from the China Atrial Fibrillation Registry, involving 52,530 hospitalized patients from 236 hospitals between February 2015 and December 2019, indicated that only 45.2% of eligible high stroke‐risk patients were administered OACs [12]. Therefore, while OAC use among Chinese AF patients is improving, substantial gaps persist.

The sharp increase in NOAC usage is noteworthy, aligning with global trends. NOACs have become the predominant choice for OACs by 2019 [8, 13]—an uptick fueled by significant price reductions post their inclusion in the National Health Insurance scheme in 2017, which further established their cost‐effectiveness compared to warfarin in non‐valvular atrial fibrillation (NVAF) scenarios [14]. The overall increase in OAC prescription rates can largely be attributed to the enhanced accessibility and affordability of NOACs [13]. This underscores the necessity for a comprehensive and updated analysis of OAC utilization trends, which is vital for understanding their current status and implications within healthcare settings.

Central to effective AF management are physicians, who play a crucial role in delivering extensive counseling and education regarding anticoagulation therapy to patients [15]. Evidence suggests that adept communication by physicians facilitates patient acceptance of NOACs [16]. However, a persisting gap in OAC knowledge among physicians in China poses challenges to achieve optimal patient care [7, 15]. This knowledge deficit spans various regions and healthcare settings, undermining the quality of patient management in non‐cardiology units [7]. Much of the current research focuses on cardiology patients, indicating a scarcity of studies examining anticoagulation strategies among AF patients in other medical domains.

This study aims to bridge this knowledge gap by conducting a retrospective review of electronic health records from patients treated at hospitals affiliated with the China Atrial Fibrillation Center (AFC). Our objective is to investigate the temporal dynamics in OAC management from January 2018 to December 2023 and assess the variations in OAC treatment across cardiology, internal medicine, and surgical departments, thus offering a more holistic view of current practices and areas in need of improvement.

## 2 Results

### 2.1 Patient Characteristics

**FIGURE 1:**
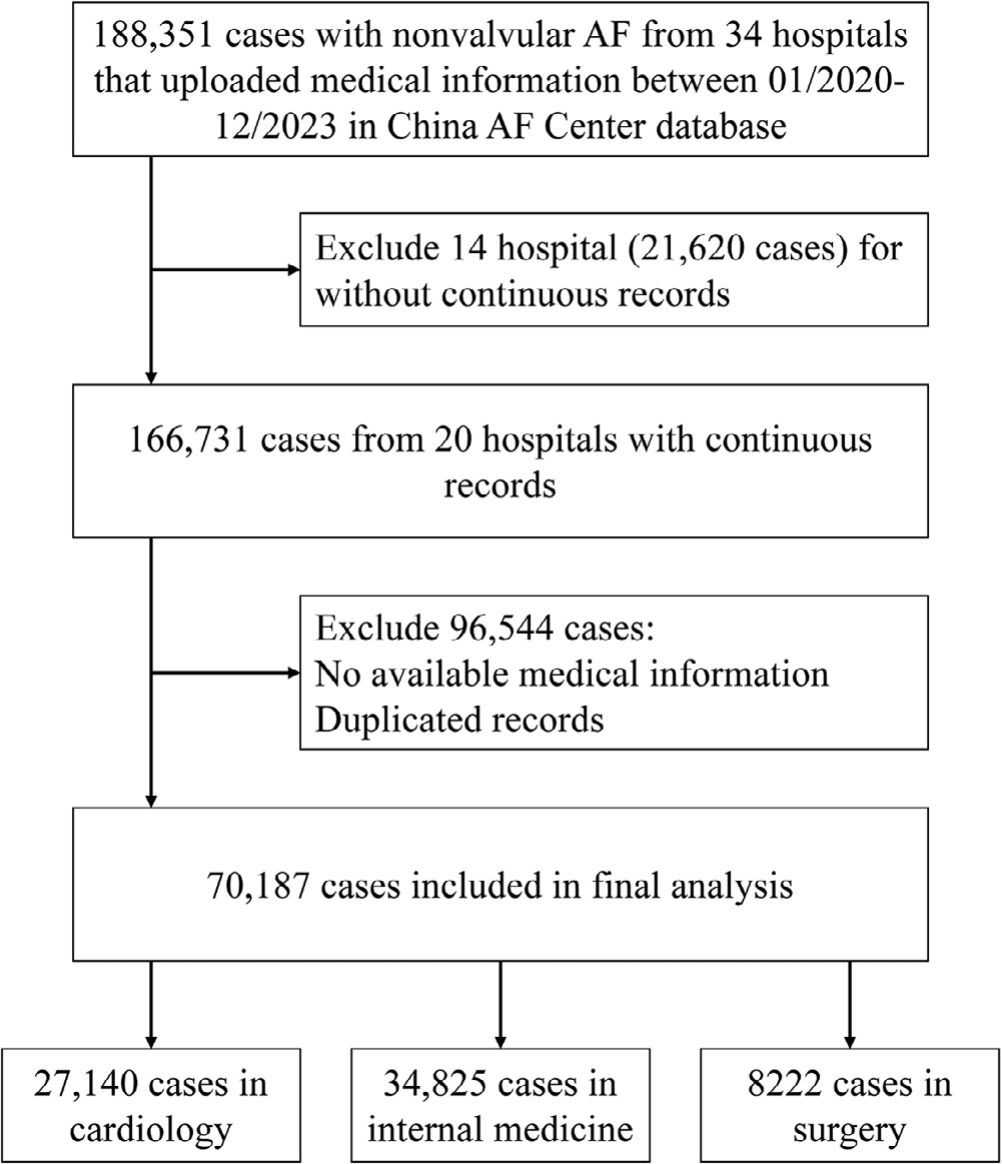
Flowchart of patient selection. NVAF, non‐valvular atrial fibrillation.

Our study encompassed 70,187 eligible patients from 20 hospitals for the final analysis, with distribution across three departments: 27,140 in cardiology, 34,825 in internal medicine, and 8222 in surgery (Figure 1). Primary patient demographics and health characteristics are detailed in Table 1. The mean age of the cohort was 72.3 (11.3) years, with individuals in internal medicine being older and those in surgery younger than the cardiology group. A total of 38,194 patients (54.41%) were male. The most prevalent comorbidity associated with AF was hypertension (50.84%), followed by a history of stroke/transient ischemic attack/systemic embolism (25.99%), and diabetes mellitus (18.73%). CHA_2_DS_2_‐VASc score was 3.24 ± 1.72. Compared with cardiology, patients in internal medicine had higher CHA_2_DS_2_‐VASc score, largely due to older age and a higher incidence of stroke/TIA/systemic embolism; furthermore, the use of upstream therapy drugs was less common in internal medicine and surgery (*p* < 0.001). Among the study sample, 34.59% were from tertiary hospitals. Baseline characteristics of patients between tertiary and non‐tertiary hospital are provided in Tables S1 and S2; these were comparable to the overall patient distribution across various departments.

**TABLE 1:** Baseline characteristics of study population according to clinical departments versus cardiology.

	Overall (*n* = 70,187)	Cardiology (*n* = 27,140)	Internal medicine (*n* = 34,825)	Surgery (*n* = 8222)
Age (years), mean (SD)	72.3 (11.3)	71 (11.4)	73.6 (10.9)^b^	70.9 (11.7)^a^
65‒74	22,366 (31.87%)	9197 (33.89%)	10,561 (30.33%)^c^	2608 (31.72%)^c^
≧75	32,400 (46.16)	11,131 (41.01%)	17,836 (51.22%)^c^	3433 (41.75%)
Male	38,194 (54.41)	14,299 (52.69)	19,140 (54.96)^c^	4755 (57.83)^c^
Congestive heart failure	13,183 (18.78)	6533 (24.07)	5960 (17.11)^c^	690 (8.39)^c^
Hypertension	35,681 (50.84)	14,072 (51.85)	17,933 (51.49)	3676 (44.71)^c^
Diabetes mellitus	13,148 (18.73)	5094 (18.77)	6780 (19.47)^a^	1274 (15.5)^c^
Stroke/TIA/systemic embolism	18,242 (25.99)	4000 (14.74)	12,584 (36.13)^c^	1658 (20.17)^c^
Vascular disease	10,006 (14.26)	3798 (13.99)	5652 (16.23)^c^	556 (6.76)^c^
CHA_2_DS_2_‐VASc score	3.24 (1.72)	3.01 (1.65)	3.54 (1.74)^c^	2.73 (1.62)^c^
0	3220 (4.59)	1512 (5.57)	1139 (3.27)	569 (6.92)
1	8404 (11.97)	3710 (13.67)	3257 (9.35)	1437 (17.48)
2	13,283 (18.93)	5574 (20.54)	5801 (16.66)	1908 (23.21)
3	15,193 (21.65)	6164 (22.71)	7194 (20.66)	1835 (22.32)
4	13,470 (19.19)	5122 (18.87)	7063 (20.28)	1285 (15.63)
5	9164 (13.06)	3062 (11.28)	5390 (15.48)	712 (8.66)
6	5126 (7.3)	1420 (5.23)	3354 (9.63)	352 (4.28)
7	1978 (2.82)	485 (1.79)	1383 (3.97)	110 (1.34)
8	331 (0.47)	85 (0.31)	233 (0.67)	13 (0.16)
9	18 (0.03)	6 (0.02)	11 (0.03)	1 (0.01)
Renal disease	5500 (7.84)	2267 (8.35)	2796 (8.03)	437 (5.32)^c^
Liver disease	2601 (3.71)	941 (3.47)	1293 (3.71)	367 (4.46)^c^
Anemia	5093 (7.26)	1545 (5.69)	2905 (8.34)^c^	643 (7.82)^c^
Bleeding history				
Cerebral hemorrhage	967 (1.38)	171 (0.63)	521 (1.5)^c^	275 (3.34)^c^
Gastrointestinal bleeding	1849 (2.63)	320 (1.18)	1336 (3.84)^c^	193 (2.35)^c^
Other bleeding diseases	1293 (1.84)	88 (0.32)	676 (1.94)^c^	529 (6.43)^c^
Hospitalization outcomes (*N*, ‰)	75 (1.07)	10 (0.37)	59 (1.69)^c^	6 (0.73)
Length of hospital stay (day)	8.47 (6.85)	7.19 (4.60)	8.79 (7.28)^c^	11.39 (9.58)^c^
Hyperthyroidism	1419 (2.02)	627 (2.31)	702 (2.02)^a^	90 (1.09)^c^
Malignant tumor	3116 (4.44)	267 (0.98)	1420 (4.08)^c^	1429 (17.38)^c^
Hemoglobin level (g/L)				
90‒120	4543 (6.47)	1765 (6.5)	2178 (6.25)	600 (7.3)^a^
60‒90	1102 (1.57)	407 (1.5)	525 (1.51)	170 (2.07)^c^
<60	178 (0.25)	61 (0.22)	100 (0.29)	17 (0.21)
Platelet (10^9^/L)				
50‒100	1043 (1.49)	376 (1.39)	484 (1.39)	183 (2.23)^c^
<50	254 (0.36)	49 (0.18)	170 (0.49)^c^	35 (0.43)^c^
ALT or AST (U/L)				
Above upper limit level	2588 (3.69)	1234 (4.55)	1223 (3.51)^c^	131 (1.59)^c^
More than three times of upper limit level	483 (0.69)	235 (0.87)	219 (0.63)^b^	29 (0.35)^c^
Creatinine (µmol/L)				
Above upper limit level	4638 (6.61)	1892 (6.97)	2317 (6.65)	429 (5.22)^c^
≥200	665 (0.95)	153 (0.56)	456 (1.31)^c^	56 (0.01)
OAC	30,153 (42.96)	16,061 (59.18)	11,630 (33.4)^c^	2462 (29.94)^c^
NOAC	22,572 (32.16)	12,397 (45.68)	8632 (24.79)^c^	1543 (18.77)^c^
Warfarin	8315 (11.85)	4086 (15.06)	3244 (9.32)^c^	985 (11.98)^c^
Antiplatelet drugs	28,440 (40.52)	11,961 (44.07)	15,407 (44.24)	1072 (13.04)^c^
Class I/III antiarrhythmic drugs	11,837 (16.86)	5670 (20.89)	4666 (13.4)^c^	1501 (18.26)^c^
β‐Blockers	36,075 (51.4)	16,977 (62.55)	15,645 (44.92)^c^	3453 (42.0)^c^
Chinese medicine	4523 (6.44)	2406 (8.87)	1889 (5.42)^c^	228 (2.77)^c^
Other antiarrhythmic drugs	13,608 (19.39)	6088 (22.43)	5283 (15.17)^c^	2237 (27.21)^c^
Statins	41,841 (59.61)	19,115 (70.43)	20,748 (59.58)^c^	1978 (24.06)^c^
SGLT2i	4147 (5.91)	2937 (10.82)	1034 (2.97)^c^	176 (2.14)^c^
ACEI/ARB	27,209 (38.77)	14,258 (52.54)	11,015 (31.63)^c^	1936 (23.55)^c^
MRAs	28,281 (40.29)	14,292 (52.66)	12,288 (35.28)^c^	1701 (20.69)^c^

Abbreviations: ACEI, angiotensin converting enzyme inhibitors; ALT, alanine transaminase; ARB, angiotensin receptor inhibitor; AST, aspartate aminotransferase; MRAs, mineralocorticoid receptor antagonists; NOAC, non‐VKA oral anticoagulants; OAC, oral anticoagulants; SGLT2i, sodium glucose cotransporter inhibitors.

^a^ *p* < 0.05.

^b^ *p* < 0.01.

^c^ *p* < 0.001.

### 2.2 Temporal Trends in the Use of OAC

**FIGURE 2:**
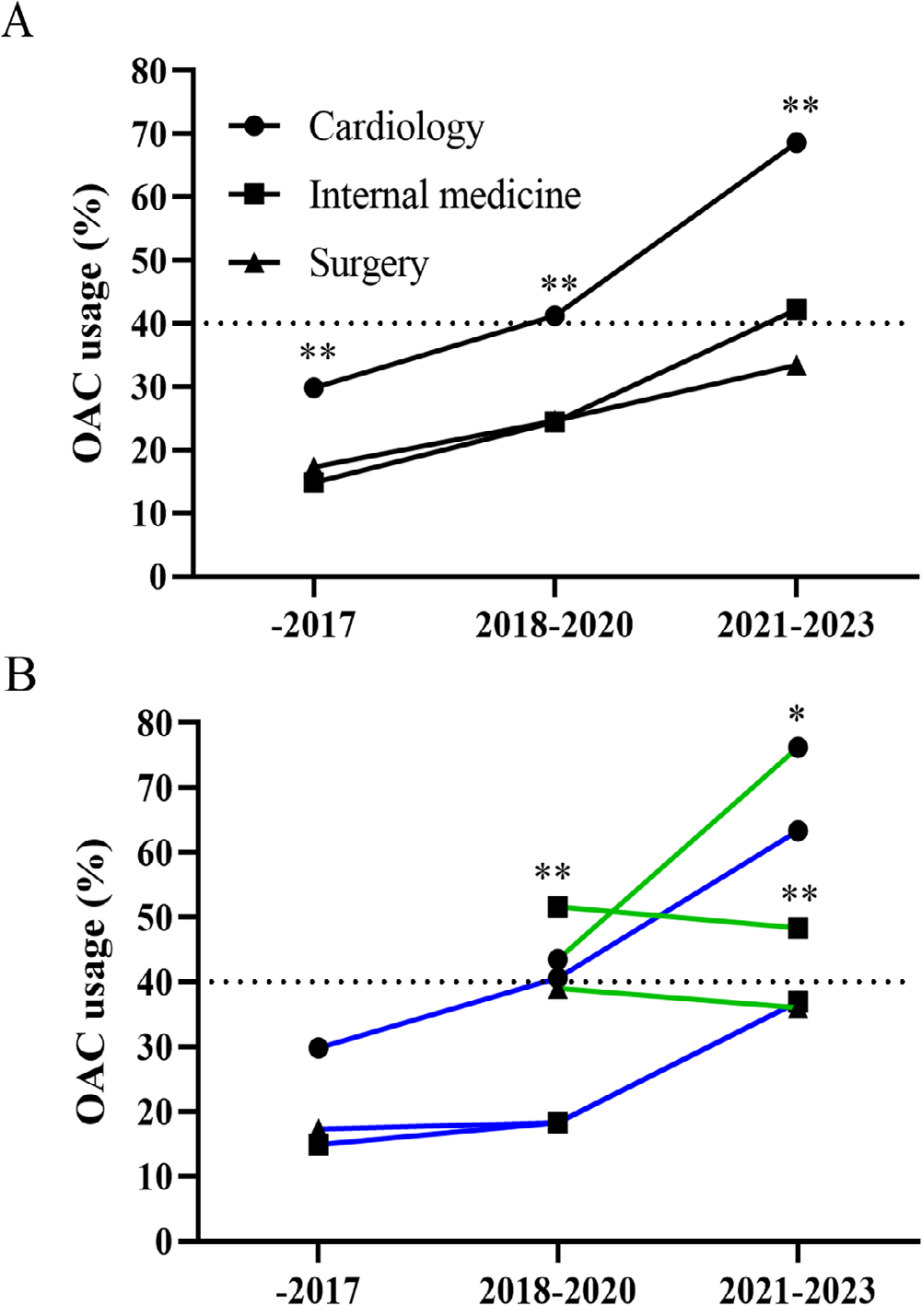
Temporal trend of anticoagulant prescription in AF patients. (A) Significant lower percentage of oral anticoagulants prescription in internal medicine or surgery compared to cardiology (^**^ *p* < 0.01) for overall patients. (B) Significant lower percentage of oral anticoagulants prescription in patients from non‐tertiary hospitals (blue line) compared to those tertiary hospitals (green line) (^**^ *p* < 0.01 for internal medicine, ^*^ *p* < 0.05 for cardiology), while not in surgery in 2021‒2023 year.

Our analysis separated the data into three distinct periods: prior to 2018, 2018‒2020, and 2021‒2023. The usage of OACs increased from 29.8% to 68.8% in cardiology, from 14.8% to 42.2% in internal medicine, and from 17.3% to 33.4% in surgery (Figure 2A and Table S3). Specifically, OAC use in cardiology departments of tertiary hospitals rose to 76.2% between 2021 and 2023, a rate significantly higher than in non‐tertiary settings. However, trends in OAC uptake within the internal medicine and surgery departments of tertiary hospitals proved unchanged after 2018. An increase in OAC prescriptions in non‐cardiology departments was more pronounced in non‐tertiary hospitals (Figure 2B and Table S3). The proportion of patients receiving OACs has been gradually increasing across different hospitals. However, this proportion is not entirely uniform (as listed in Table S4). In tertiary hospitals, the variation is minimal, whereas in non‐tertiary hospitals, there is a more notable disparity.

**FIGURE 3:**
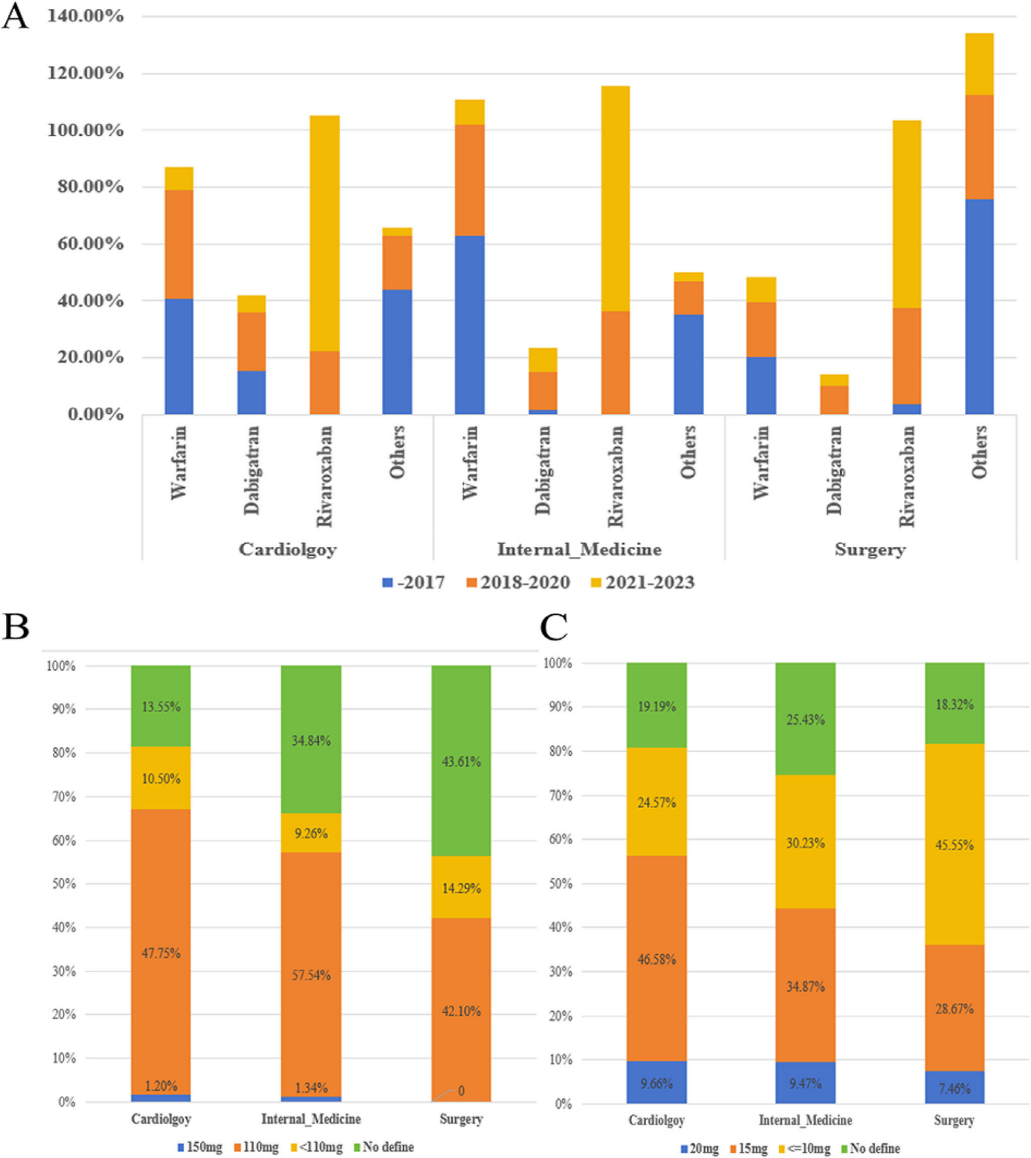
Composition of anticoagulant drugs in different departments. (A) The proportion of usage of the same medication across different time periods within the same apartment. The use of warfarin has progressively decreased, while the use of rivaroxaban has significantly increased, with the highest proportion. (B) Doses of dabigatran. (C) Doses of rivaroxaban.

With respect to specific OAC, before 2018, warfarin predominated prior to 2018, but its dominance waned by the 2021‒2023 period. Post‐2018, rivaroxaban emerged as the increasingly favored choice, accounting for approximately 80% of OAC prescriptions by the latest period (Figure 3A and Table S5). Contrarily, the proportion of dabigatran use not only stalled after 2020 but showed a decline. For patients prescribed dabigatran, less than 1.5% used the standard dose of 150 mg twice daily (Figure 3B). In terms of rivaroxaban doses, less than 10% of patients across the studied departments were on the 20 mg daily regimen; the majority received a daily dose of 15 mg in cardiology (46.58%) and internal medicine (34.87%), while in surgery, a dose of 10 mg or less was more common (Figure 3C).

To explore the sex‐based differences in use of OAC over time, we compared the trend of OAC prescription in our study. As shown in Figure S1, the usage rates of OAC among both males and females in cardiology, internal medicine, and surgery have shown a gradual upward trend over time. During the same time period, in surgery, the OAC usage rate among males is significantly lower than that of females (*p* < 0.05), while in cardiology and other internal medicine, the difference is not statistically significant.

### 2.3 Oral Anticoagulants Use in Patients With High Risk of Stroke

**FIGURE 4:**
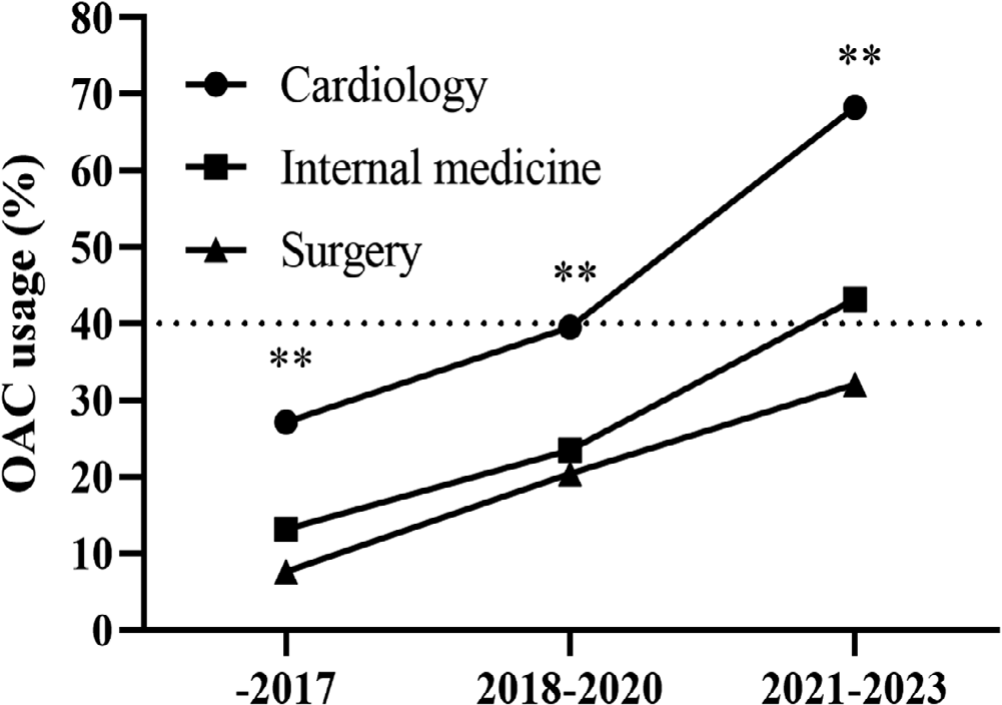
Temporal trend of anticoagulant prescription in AF patients with high risk of stroke. Significant lower percentage of oral anticoagulants prescription in internal medicine or surgery compared to cardiology (^**^ *p* < 0.01).

In our cohort, 54,343 patients (77.4%) were considered at high risk for stroke. The OAC prescription rate pre‐2018 was a mere 27.2% in cardiology, 13.2% in internal medicine, and 7.7% in surgery. These rates have seen a significant uptick in recent years (Figure 4 and Table S6). During the years 2021‒2023, OAC therapy rates matched those observed in patients with a moderate risk profile for stroke within the corresponding departments. Nevertheless, OAC prescription rates remained below 50% in internal medicine and surgery (Figure 4 and Table S6). A gradual decline in warfarin use paralleled a swift rise in rivaroxaban uptake.

### 2.4 In‐Hospital Outcomes

We assessed both the duration of hospitalization and mortality status at discharge. Compared to the cardiology department, patients in non‐cardiology settings experienced significantly longer hospital stays (*p* < 0.001, Table 1). OAC treatment was also associated with prolonged hospitalization compared to non‐OAC patients (Table S7). Overall, in‐hospital mortality was 1.07‰, with internal medicine reporting significantly higher mortality rates than cardiology. Mortality tended to be lower among patients undergoing OAC therapy, and the difference in unadjusted mortality rates between OAC and non‐OAC therapy patients from internal medicine was statistically significant (Table 1 and Table S7). In the multivariable logistic analysis of factors associated with in‐hospital death (Table 2), OAC therapy was identified as a protective factor, whereas the department of treatment was not found to be an independent factor.

**TABLE 2:** Logistic regression analysis for factors associated with in‐hospital death (*p* < 0.05 in univariable regression into multivariable model).

Variables	Univariable regression		Multivariable regression	
	OR (95% CI)	*p*‐Value	OR (95% CI)	*p*‐Value
Age	1.707 (1.221‒2.385)	0.002	1.185 (0.796‒1.764)	0.404
Female	1.160 (0.740‒1.816)	0.518		
Department	1.619 (1.160‒2.261)	0.005	1.011 (0.689‒1.484)	0.956
Heart failure	3.592 (2.279‒5.662)	<0.001	3.483 (2.075‒5.846)	<0.001
Hypertension	0.719 (0.455‒1.137)	0.159		
Diabetes	0.910 (0.500‒1.654)	0.756		
Stroke	1.032 (0.613‒1.737)	0.906		
Systemic embolism	0.564 (0.078‒4.056)	0.569		
Coronary heart disease	1.570 (0.782‒3.151)	0.205		
Other artery disease	0.802 (0.293‒2.196)	0.667		
CHA_2_DS_2_‐VASc score	1.227 (1.079‒1.395)	0.002	1.256 (1.055‒1.496)	0.010
Hyperthyroidism	2.021 (0.636‒6.423)	0.233		
Malignant tumor	1.873 (0.813‒4.318)	0.141		
Anemia	2.684 (1.475‒4.884)	0.001	1.739 (0.945‒3.201)	0.076
Renal disease	2.024 (1.067‒3.839)	0.031	1.103 (0.560‒2.172)	0.777
Liver disease	3.109 (1.492‒6.479)	0.002	2.307 (1.069‒4.979)	0.033
Oral anticoagulants	0.136 (0.063‒0.297)	<0.001	0.183 (0.081‒0.413)	<0.001
Antiplatelet drugs	0.252 (0.133‒0.478)	<0.001	0.369 (0.176‒0.775)	0.008
Class I/III antiarrhythmic drugs	3.480 (2.197‒5.512)	<0.001	3.892 (2.419‒6.263)	<0.001
β‐Blockers	0.392 (0.238‒0.645)	<0.001	0.567 (0.335‒0.960)	<0.001
Mineralocorticoid receptor antagonists	0.502 (0.299‒0.846)	0.010	0.524 (0.294‒0.933)	0.028
Statins	0.183 (0.106‒0.319)	<0.001	0.370 (0.192‒0.713)	0.003
Sodium glucose cotransporter inhibitors	0.215 (0.030‒1.547)	0.127		
Angiotensin converting enzyme or angiotensin receptor inhibitors	0.498 (0.293‒0.847)	0.010	1.029 (0.579‒1.830)	0.922

### 2.5 Factors Influencing Anticoagulation Therapy

**FIGURE 5:**
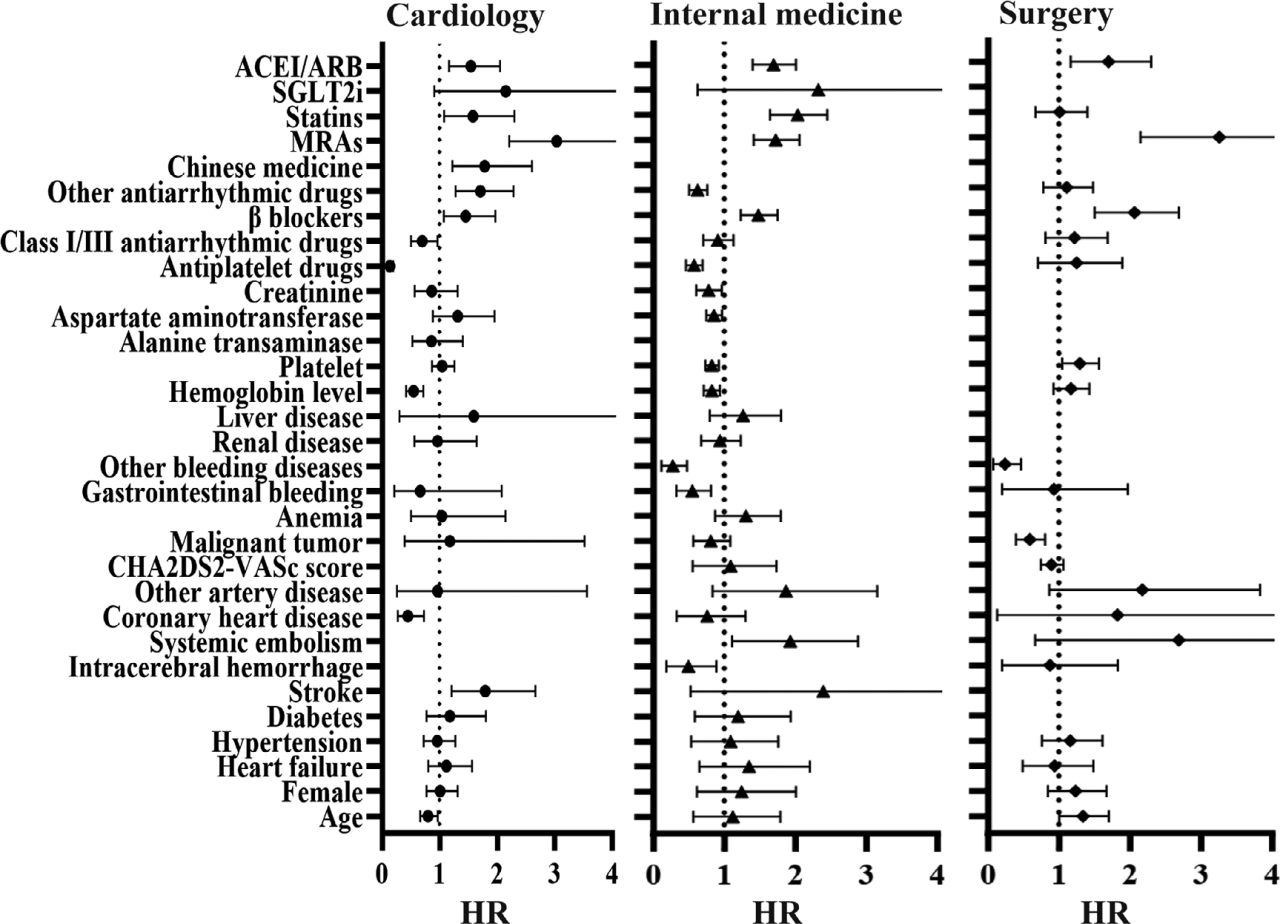
Forest plot of factors association with OAC prescription for different departments. ACEI, angiotensin converting enzyme inhibitors; ARB, angiotensin receptor blockers; MRAs, mineralocorticoid receptor antagonists; SGLT2i, sodium glucose cotransporter inhibitors.

A logistic regression model was employed to identify factors influencing OAC use among AF patients. As listed in Table 3, many of the influencing factors studied met the criteria for inclusion in the multivariate regression model. According to our multivariable regression analysis, determinants of OAC use varied by department (Table 4 and Figure 5). A history of bleeding, lower hemoglobin levels, and concurrent antiplatelet therapy were associated with reduced OAC use. Advanced age and coronary heart disease were notably influential for OAC use within the cardiology department. Additionally, malignancy presented a barrier to OAC use in surgical patients. While the individual components of the CHA_2_DS_2_‐VASc score were considerable in univariate analysis, most did not retain significance in the multivariate model. Upstream treatments, such as ACE inhibitors/ARBs, mineralocorticoid receptor antagonists, and β‐blockers, demonstrated a uniformly positive influence on the acceptance of OAC across all clinical departments.

**TABLE 3:** Univariable regression analysis for factors association with OAC prescription for different departments.

Variables	Cardiology		Internal medicine		Surgery	
	OR (95% CI)	*p*‐Value	OR (95% CI)	*p*‐Value	OR (95% CI)	*p*‐Value
Age	0.897 (0.870‒0.925)	<0.001	0.880 (0.855‒0.906)	<0.001	0.700 (0.660‒0.742)	<0.001
Female	1.082 (1.031‒1.136)	0.001	1.082 (1.035‒1.132)	<0.001	1.469 (1.335‒1.615)	<0.001
Heart failure	1.176 (1.111‒1.245)	<0.001	1.338 (1.263‒1.417)	<0.001	2.461 (2.102‒2.880)	<0.001
Hypertension	1.099 (1.047‒1.153)	<0.001	1.171 (1.119‒1.224)	<0.001	0.873 (0.794‒0.960)	<0.001
Diabetes	1.095 (1.029‒1.166)	0.004	1.237 (1.171‒1.308)	<0.001	0.921 (0.807‒1.050)	0.219
Stroke	1.397 (1.300‒1.500)	<0.001	1.278 (1.221‒1.339)	<0.001	0.969 (0.850‒1.105)	0.640
Intracerebral hemorrhage	0.841 (0.622‒1.138)	0.262	0.626 (0.511‒0.766)	<0.001	0.177 (0.112‒0.279)	<0.001
Systemic embolism	1.227 (0.688‒2.187)	0.488	1.846 (1.653‒2.062)	<0.001	3.375 (2.647‒4.303)	<0.001
Coronary heart disease	0.370 (0.342‒0.399)	<0.001	0.724 (0.658‒0.797)	<0.001	0.622 (0.457‒0.849)	0.003
Other artery disease	1.783 (1.521‒2.091)	<0.001	1.580 (1.472‒1.696)	<0.001	1.523 (1.212‒1.915)	<0.001
CHA_2_DS_2_‐VASc score	1.003 (0.988‒1.017)	0.721	1.064 (1.050‒1.077)	<0.001	0.965 (0.937‒0.994)	0.018
Hyperthyroidism	0.935 (0.796‒1.097)	0.409	0.978 (0.834‒1.146)	0.781	0.899 (0.565‒1.429)	0.652
Malignant tumor	1.408 (1.089‒1.819)	<0.001	0.595 (0.525‒0.674)	<0.001	0.494 (0.429‒0.569)	<0.001
Anemia	0.842 (0.759‒0.934)	0.001	0.649 (0.595‒0.707)	<0.001	1.012 (0.849‒1.206)	0.896
Gastrointestinal bleeding	0.372 (0.296‒0.469)	<0.001	0.362 (0.312‒0.421)	<0.001	0.279 (0.177‒0.441)	<0.001
Other bleeding diseases	0.755 (0.496‒1.148)	0.188	0.425 (0.348‒0.518)	<0.001	0.163 (0.117‒0.229)	<0.001
Renal disease	1.132 (1.036‒1.236)	0.006	1.113 (1.027‒1.207)	0.009	1.146 (0.933‒1.408)	0.193
Liver disease	1.630 (1.415‒1.877)	<0.001	1.227 (1.094‒1.376)	<0.001	1.043 (0.831‒1.308)	0.717
Hemoglobin level	0.761 (0.708‒0.818)	<0.001	0.783 (0.731‒0.839)	<0.001	1.229 (1.067‒1.417)	0.004
Platelet	0.802 (0.733‒0.879)	<0.001	0.773 (0.721‒0.828)	<0.001	1.190 (1.054‒1.342)	0.005
Alanine transaminase	0.885 (0.793‒0.988)	0.029	0.898 (0.778‒1.037)	0.143	0.781 (0.433‒1.408)	0.411
Aspartate aminotransferase	0.826 (0.743‒0.919)	<0.001	0.844 (0.743‒0.958)	0.009	0.768 (0.467‒1.263)	0.298
Creatinine	0.871 (0.800‒0.948)	0.001	0.747 (0.695‒0.802)	<0.001	0.915 (0.766‒1.092)	0.325
Antiplatelet drugs	0.322 (0.306‒0.339)	<0.001	0.676 (0.646‒0.707)	<0.001	1.435 (1.255‒1.641)	<0.001
Class I/III antiarrhythmic drugs	0.937 (0.883‒0.995)	0.032	0.768 (0.717‒0.822)	<0.001	2.069 (1.844‒2.322)	<0.001
β‐Blockers	1.487 (1.415‒1.563)	<0.001	2.013 (1.924‒2.106)	<0.001	2.727 (2.475‒3.005)	<0.001
Other antiarrhythmic drugs	1.408 (1.327‒1.495)	<0.001	0.809 (0.759‒0.862)	<0.001	1.795 (1.620‒1.989)	<0.001
Chinese medicine	0.841 (0.773‒0.915)	<0.001	0.966 (0.875‒1.066)	0.487	0.994 (0.746‒1.326)	0.968
Mineralocorticoid receptor antagonists	1.306 (1.244‒1.371)	<0.001	1.532 (1.463‒1.604)	<0.001	4.070 (3.641‒4.550)	<0.001
Statins	1.206 (1.144‒1.271)	<0.001	1.990 (1.898‒2.086)	<0.001	2.222 (2.000‒2.470)	<0.001
Sodium glucose cotransporter inhibitors	3.157 (2.872‒3.471)	<0.001	3.125 (2.753‒3.546)	<0.001	4.462 (3.263‒6.102)	<0.001
Angiotensin converting enzyme or angiotensin receptor inhibitors	1.576 (1.501‒1.654)	<0.001	1.932 (1.844‒2.025)	<0.001	2.456 (2.209‒2.731)	<0.001

**TABLE 4:** Multivariable regression analysis for factors association with OAC prescription for different departments (*p* < 0.05 in Table 3 into multivariable model).

Variables	Cardiology		Internal medicine		Surgery	
	OR (95% CI)	*p*‐Value	OR (95% CI)	*p*‐Value	OR (95% CI)	*p*‐Value
Age	0.799 (0.662‒0.965)	0.020	1.000 (0.560‒1.785)	0.999	1.305 (1.000‒1.704)	0.050
Female	1.009 (0.774‒1.314)	0.949	1.108 (0.612‒2.006)	0.734	1.186 (0.843‒1.669)	0.326
Heart failure	1.118 (0.800‒1.562)	0.515	1.194 (0.648‒2.202)	0.570	0.848 (0.485‒1.484)	0.564
Hypertension	0.958 (0.720‒1.275)	0.767	0.968 (0.534‒1.756)	0.915	1.105 (0.758‒1.612)	0.603
Diabetes	1.180 (0.773‒1.802)	0.442	1.062 (0.583‒1.933)	0.844		
Stroke	1.794 (1.209‒2.661)	0.004	1.618 (0.521‒5.031)	0.405		
Intracerebral hemorrhage			0.403 (0.183‒0.886)	0.024	0.599 (0.196‒1.827)	0.367
Systemic embolism			1.786 (1.106‒2.883)	0.018	1.906 (0.662‒5.487)	0.232
Coronary heart disease	0.445 (0.274‒0.725)	0.001	0.651 (0.326‒1.299)	0.224	0.76 (0.126‒4.587)	0.765
Other artery disease	0.960 (0.259‒3.558)	0.951	1.620 (0.832‒3.153)	0.156	1.818 (0.862‒3.834)	0.117
CHA_2_DS_2_‐VASc score			0.979 (0.553‒1.733)	0.942	0.887 (0.741‒1.062)	0.191
Malignant tumor	1.177 (0.394‒3.517)	0.770	0.777 (0.560‒1.080)	0.133	0.561 (0.392‒0.803)	0.002
Anemia	1.038 (0.503‒2.142)	0.919	1.246 (0.867‒1.790)	0.234		
Gastrointestinal bleeding	0.663 (0.212‒2.078)	0.481	0.511 (0.321‒0.813)	0.005	0.624 (0.198‒1.967)	0.421
Other bleeding diseases			0.230 (0.112‒0.474)	<0.001	0.184 (0.074‒0.461)	<0.001
Renal disease	0.960 (0.562‒1.640)	0.881	0.909 (0.673‒1.228)	0.535		
Liver disease	1.592 (0.300‒8.438)	0.585	1.192 (0.79‒1.798)	0.402		
Hemoglobin level	0.547 (0.419‒0.715)	<0.001	0.816 (0.708‒0.939)	0.005	1.148 (0.923‒1.429)	0.216
Platelet	1.043 (0.866‒1.257)	0.657	0.821 (0.731‒0.922)	0.001	1.275 (1.043‒1.560)	0.018
Alanine transaminase	0.859 (0.526‒1.401)	0.542				
Aspartate aminotransferase	1.310 (0.881‒1.95)	0.183	0.848 (0.743‒0.967)	0.014		
Creatinine	0.861 (0.566‒1.310)	0.484	0.761 (0.603‒0.961)	0.022		
Antiplatelet drugs	0.137 (0.099‒0.190)	<0.001	0.563 (0.459‒0.691)	<0.001	1.152 (0.701‒1.894)	0.576
Class I/III antiarrhythmic drugs	0.696 (0.504‒0.963)	0.029	0.890 (0.701‒1.129)	0.337	1.166 (0.807‒1.684)	0.413
β‐Blockers	1.452 (1.072‒1.965)	0.016	1.464 (1.225‒1.749)	<0.001	2.008 (1.501‒2.685)	<0.001
Other antiarrhythmic drugs	1.708 (1.277‒2.284)	<0.001	0.616 (0.502‒0.755)	<0.001	1.069 (0.775‒1.475)	0.686
Chinese medicine	1.782 (1.222‒2.600)	0.003				
Mineralocorticoid receptor antagonists	3.032 (2.205‒4.168)	<0.001	1.703 (1.411‒2.055)	<0.001	3.114 (2.146‒4.519)	<0.001
Statins	1.576 (1.079‒2.300)	0.019	2.004 (1.642‒2.446)	<0.001	0.965 (0.667‒1.397)	0.851
Sodium glucose cotransporter inhibitors	2.147 (0.907‒5.083)	0.082	1.702 (0.624‒4.64)	0.299	3.015 (0.700‒12.976)	0.138
Angiotensin converting enzyme or angiotensin receptor inhibitors	1.544 (1.162‒2.050)	0.003	1.676 (1.399‒2.007)	<0.001	1.634 (1.161‒2.298)	0.005

## 3 Discussion

This study unveiled several key findings: a progressive increase in the prescription of OACs throughout the study period; marked disparities in OAC prescription rates among various hospital departments; notably, the OAC prescription rates for patients at high risk of stroke remained suboptimal, particularly within the internal medicine and surgery departments. This research emphasizes the variation in OAC prescription rates across different hospital departments.

OACs are recognized as the standard care for stroke prevention in patients with AF. However, in China, OACs, especially NOACs, are under‐prescribed. A community‐based survey involving 47,841 adults aged 45 years or older across seven geographical regions of China between 2014 and 2016 reported that the prevalence of AF was 1.8%, estimated to be 7.9 million patients. Yet, only 6.0% of those with high‐risk AF received anticoagulation therapy [17]. The GLORIA‐AF phase II and III data demonstrated a continued increase in OAC prescription over time (from 21.0% to 41.0%) among Chinese patients with AF [6]. Despite this increase, the anticoagulation landscape for stroke prevention in AF patients in China remains suboptimal when compared to Asia (61.6%) and globally (82.2%) [18]. A nationwide cohort study conducted from November 2017 to October 2018 in 362 tertiary general medical centers revealed an elevated OAC prescription rate of 79.1% [11]. In contrast, the most recent data from the China‐Atrial Fibrillation Registry, encompassing 52,530 hospitalized patients from 236 hospitals between February 2015 and December 2019, indicated that only 45.2% of qualified high‐risk stroke patients received OACs [12, 19]. Our research, based on electronic medical records, aims to minimize errors associated with manual reporting; the study encompasses both tertiary and non‐tertiary hospitals. We observed a significant uptick in anticoagulation rates over time, especially in the past 3 years, with cardiology departments in tertiary hospitals reaching a therapy rate of 76.2%, and 63.4% in non‐tertiary hospitals. The use of OACs among patients in the cardiology department of tertiary hospitals has significantly increased, but there is still a considerable gap compared to the use rate in Japan (90.3%) and non‐Asian countries (86.7%) from GLORIA‐AF phase II investigated in 2014‒2016 [7]. This trend is concerning, particularly among high‐risk populations, where insufficient anticoagulation rates are prevalent [9]. Previous studies [6, 20] corroborate our observation that anticoagulation rates in patients with AF at high risk of stroke do not surpass those not at high risk, a phenomenon also noted in our research. In this study, hypertension was the most common comorbidity, followed by stroke. Managing anticoagulation in stroke patients poses a significant challenge in clinical practice. The concurrent high risk of bleeding is also a reason that hampers OACs use. Although NOAC help to reduce the risk of bleeding [21], a high risk of bleeding should not be an absolute contraindication for the use of OACs [1]. However, it remains an urgent issue to be addressed in clinical practice.

Previous research predominantly showed prescriptions being issued by cardiologists [6]. Even in observations from neurology departments also highlighted insufficient OAC therapy [9]. Despite a noticeable increase in anticoagulation rates across different departments over time, rates in medical and surgical departments remain markedly lower than those observed under the care of cardiology departments. Our findings indicated that the factors influencing anticoagulant therapy are varied across departments, highlighting the need to address specific barriers to anticoagulation faced by various departments. For example, in neurology, the rate of anticoagulation among patients with concomitant cerebral infarction remains low [9]. There is still no consensus on the duration of anticoagulation for patients with AF who have acute cerebrovascular diseases [22]. Additionally, in surgery, issues such as the choice of perioperative anticoagulation regimens, postoperative AF anticoagulation plans, and anticoagulation strategies for patients with malignancies are still unclear [23]. Complex situations, gaps of OAC knowledge, and physicians' effective communication may all attribute to the gap of OAC use in different departments. The types of anticoagulants used do not significantly differ from those used in cardiology, yet the rates in other departments remain below 50%, there is considerable room for improvement. Importantly, the rate of anticoagulation in non‐cardiology departments at tertiary hospitals appears stagnant, suggesting the necessity of providing AF management guidance to physicians outside the cardiology department.

Another aspect warranting attention is the scant evidence on the efficacy of OAC in AF patients outside cardiology research, particularly in specialized departments such as ICU [24] and surgery [25]. Our study discovered that unadjusted mortality rates were lower in the anticoagulated group of internal medicine patients with AF. In the logistic regression analysis, treatment in different departments was not an independent risk factor for in‐hospital mortality. However, the use of anticoagulant medication was an independent factor associated with reduced risk of in‐hospital mortality. The data are not yet sufficient to draw definitive conclusions. Future targeted research is required to elucidate the potential reasons and the true role of anticoagulation.

Previous studies have revealed a significant clinical gap in anticoagulation outcomes between our country and other regions such as Asia, Europe, and America [6, 7, 8]. Factors contributing to low anticoagulation rates include high costs and lack of health insurance coverage, deterring the persistence of NOACs in China [26, 27]. In contrast, NOACs promote OAC prescription in Europe [26, 28]. In China, the reimbursement mechanisms for NOAC have progressively improved over the years [19]. Before 2018, the cost of novel oral anticoagulants (NOACs) was quite high, and they were not eligible for reimbursement. As we have shown in Table S8, after several negotiations by the healthcare authorities post‐2018, the price of NOACs significantly decreased and partial reimbursement became available through insurance. Furthermore, the introduction of generics from 2023 onwards has further reduced the cost of using NOACs. These changes have made NOACs more affordable and accessible to a broader patient population, contributing to an increase in their prescription. This corresponds with the changes in anticoagulation rates that we have observed. In comparison, the reimbursement policies in other Asian countries, such as Japan, have historically been more supportive of NOAC use, reflecting in their higher rates of OAC prescription. In Japan, for instance, NOACs have been included in the national health insurance coverage for a longer period, which has facilitated their widespread use. Studies conducted before 2020 lack information on the current anticoagulation status in AF patients following the widespread use of NOACs, health insurance policy modifications, and price adjustments [7, 12, 13, 19]. Our study confirms that NOAC usage has significantly increased across all departments. The usage of Warfarin in NVAF is declining, paralleled by an increasing rate of NOAC usage. Hence, the promotion of NOACs may further augment the anticoagulation rate among the Chinese population with AF. The updates and promotion of AF guidelines are also important factors. In recent years, guidelines for AF management have been updated every 3‒4 years in Europe, the United States, and China, providing an evidence‐based framework for clinicians to optimize patient care [1, 2, 3, 4]. In China, the AF management guidelines were revised in 2015, 2018, 2021, and most recently in 2024, incorporating advancements in treatment standards and risk stratification tools for stroke prevention [3]. These updates have improved the standardization of AF diagnosis and treatment practices. The latest updates emphasize stroke risk assessment using established tools such as the CHA_2_DS_2_‐VASc score and recommend NOACs as preferred agents over warfarin due to their superior safety and efficacy profiles. This guidance has helped clinicians make informed decisions and has undoubtedly contributed to increased OAC usage [3]. Furthermore, the establishment of specialized AF centers across China since 2016 (https://www.china‐afc.org/) and the formation of AF alliances in various provinces and cities have bolstered efforts to implement guideline recommendations. These initiatives have provided additional training and resources for clinicians, facilitated interdepartmental collaboration, ensured better follow‐up care for patients, and promoted the adoption of standardized treatment protocols. The dissemination of guidelines and clinician education through these networks has significantly enhanced guideline adherence, improving anticoagulation rates for patients with AF. Moreover, the differences among various departments indicate that there is substantial potential for the development of AF diagnosis and treatment outside of cardiology.

An optimal OAC dosage can maximize its effect and minimize adverse events. Yet, studies have indicated that less than 50% of patients on OAC therapy were prescribed appropriate NOAC doses in China according to recommended guidelines [6, 8]. In the current study, it is alarming that recommended NOAC doses were prescribed in less than 10% of cases. This trend was consistent across both tertiary and non‐tertiary hospitals. Our research found that the utilization of rivaroxaban has significantly increased since 2018, reaching 80% between 2021 and 2023. This is consistent with the survey results from Gong et al. conducted in five major cities in China in 2019 [13]. Studies have indicated that both dabigatran and rivaroxaban can effectively reduce thromboembolic events in Asian populations; however, compared to warfarin, rivaroxaban does not appear to lower bleeding risk [21]. The dominance of rivaroxaban may be attributed to its once‐daily dosing regime, but more crucially it might relate to factors such as price and health insurance policies [14]. Ongoing national multicenter studies examining real‐world data on the rationality of NOAC prescription in AF patients in China may shed light on the factors contributing to inappropriate NOAC usage [29, 30].

Furthermore, our study found that the utilization of upstream therapeutic drugs, including statins, ACE inhibitors/ARBs, MRBs, and symptom‐controlling medications such as β‐blockers, serves as a positive factor in promoting anticoagulant use. The Atrial Fibrillation Better Care (ABC) pathway, proposed as an integrated approach to improve AF patient management, has been associated with reduced risk for adverse outcomes in a large contemporary cohort of Asian AF patients [1]. In a large contemporary cohort of Asian patients with AF, adherence to ABC pathway was associated with a reduction of the risk for adverse outcomes [31]. Consequently, comprehensive management of AF to alleviate symptoms and enhance medication adherence also represents a crucial avenue for improving anticoagulation treatment

### 3.1 Limitations

Despite this study's strength in employing large sample, real clinical data from China, it presents several limitations. Primarily, its retrospective and observational nature, based on electronic medical records from hospitals registered at the China AFC, introduces a potential selection bias. The data might not adequately represent all population subsets, particularly those with less severe AF who might not regularly seek hospital care or those treated in non‐registered facilities. Additionally, while including data from both tertiary and non‐tertiary hospitals, there is variability among the individual hospitals within tertiary and non‐tertiary categories, highlighting significant differences in AF anticoagulation management across these hospitals. The study might not fully capture the diversity of medical practices across different Chinese regions. Disparities in healthcare access, care quality, and physician adherence to guidelines between rural and urban settings or various provinces could affect study outcomes but are not extensively analyzed. The peri‐operative period presents unique circumstances where AF may develop or be exacerbated, impacting the choice and management of oral anticoagulation. The continuity of care and follow‐up for these patients can indeed vary based on whether cardiologists, internists, or surgeons manage their treatment. This indicates that anticoagulation therapy for surgical patients with AF is complex, and addressing the low anticoagulation rates requires the collaborative efforts of a multidisciplinary team. Furthermore, data on comorbidities and outcomes were collected from medical record front pages, excluding specific documents, electrocardiograms, and other data. This limitation hinders our ability to discern AF type and whether it is newly diagnosed, affecting our judgment of the disease's impact on OAC use. Last, although the study employs multivariate logistic regression to identify factors associated with OAC prescription, unmeasured confounding variables could influence these outcomes. Variations in patient compliance, physician prescribing habits not captured by department type, or socioeconomic factors affecting healthcare and medication access are not thoroughly examined, potentially significantly influencing the interpretation of departmental or hospital anticoagulation therapy rates.

## 4 Conclusion

In conclusion, our study emphasizes the persistent underutilization of OACs in China, particularly in non‐cardiology departments, despite notable improvements in recent years. The study identifies key impediments, including advanced age, bleeding history, and treatment outside cardiology departments, while recognizing that upstream therapies tend to encourage anticoagulation acceptance. These findings suggest that comprehensive management strategies, including better integrated care pathways and enhanced physician education across specialties, are crucial for optimizing anticoagulation therapy in AF patients.

## 5 Materials and Methods

### 5.1 Study Design

This study was conducted as a multicenter, observational, retrospective cohort study involving multiple hospitals participating in the Chinese Cardiovascular Association (CCA) Database‐AFC registry (https://www.china‐afc.org/) [32]. The study period spanned from January 2015 to December 2023. Ethical approval was secured from the Institutional Committee on Human Research at Qilu Hospital (Qingdao) of Shandong University (approval no. KYLL‐2024017). Due to the retrospective nature of the study and the use of anonymized electronic medical records, the requirement for written informed consent was waived.

### 5.2 Study Population

The study included patients aged 18 years or older with a confirmed diagnosis of NVAF. Exclusion criteria were lack of comprehensive medical documentation or duplicate records. Data were collected from hospitals contributing medical records to the AFC for at least one continuous year during the study period.

### 5.3 Data Collection

Medical records were extracted from the AFC database, ensuring data consistency and completeness. The demographic data included age, sex, and race. Detailed medical histories were obtained, including previous cardiovascular events, comorbidities, and ongoing medications.

### 5.4 Disease Classification and Comorbidities

AF diagnoses and other disease categories in the electronic medical records were confirmed using International Classification of Diseases codes. Comorbidities and in‐hospital results were extracted manually from the leading pages of the medical records.

### 5.5 Laboratory Measurements

Laboratory metrics were sourced from electronic health records and included hemoglobin, platelet count, alanine aminotransferase (ALT), aspartate aminotransferase (AST), and creatinine levels. Laboratory measurements were categorized as follows—hemoglobin: normal, mild reduction (90‒120 g/L), moderate reduction (60‒90 g/L), and severe reduction (<60 g/L). Platelet count: normal (>100 × 10^9^/L), mild reduction (50‒100 × 10^9^/L), and severe reduction (<50 × 10^9^/L). ALT and AST: normal, above the upper limit of normal, more than three times the upper limit of normal. Creatinine: normal, mild increase (above the upper normal limit but <200 µmol/L), and severe increase (>200 µmol/L). In cases where multiple laboratory values were available, the most critical values were selected for analysis: the lowest values for hemoglobin and platelets, and the highest values for ALT, AST, and creatinine.

### 5.6 Stroke Risk Assessment

Stroke risk was assessed using the CHA_2_DS_2_‐VASc score, which assigns points based on patient characteristics: congestive heart failure, hypertension, age (≥75 years), diabetes mellitus, stroke/TIA/thromboembolism, vascular disease, age (65‒74 years), and sex category (female). A score of 2 or higher for males and 3 or higher for females indicated high stroke risk [1].

### 5.7 Statistical Analysis

Analyses of statistics were performed with IBM SPSS Statistics software (version 22.0, IBM Corp., Armonk, NY, USA). For hypotheses testing that were two‐sided, a *p*‐value below 0.05 was deemed statistically significant. Continuous variables were summarized as mean ± standard deviation (SD), while categorical variables were presented as frequencies and percentages. The comparison of means or medians utilized Student's *t*‐test or the Wilcoxon rank‐sum test, and chi‐square tests or Fisher's exact test were used for comparing categorical data in small samples, as suitable. The *z*‐test was used for comparing mortality rates. Factors linked to OAC prescription and in‐hospital death were identified using a multivariate logistic regression that employed a stepwise method, setting entrance and retention in the model at *p*‐values of 0.10 and 0.05, respectively. Data were graphically represented using GraphPad Prism version 8.

## Author Contributions

M.J.L. and H.H. conceptualized and undertook the research, acquired and interpreted the data, and developed the initial draft of the manuscript. J.T.W., H.S., X.S.X., Y.Z., and W.Q.H. contributed to the research execution and data acquisition. M.C. and K.D. acquired and analyzed the data. Y.C.W., B.A.Y., and G.H.Y. oversaw research and reviewed data. J.Q.Z. and C.X.H. were involved in the research design and execution, reviewed data, and obtained fundings. All authors have reviewed and confirmed the final version of the manuscript.

## Funding

The study was supported by the Natural Science Foundation of China (81970282 and 82270331) and Qingdao Key Clinical Specialty Elite Discipline (QDZDZK‐2022008), which were obtain by JQZ. The sources of funding were not involved in any aspect of the study, including its design and execution; the gathering, handling, analysis, and elucidation of data; or the drafting, revision, and endorsement of the manuscript. Additionally, they did not influence the choice to submit the manuscript for publication.

## Ethics Statement

Ethical approval was secured from the Institutional Committee on Human Research at Qilu Hospital (Qingdao) of Shandong University (approval no. KYLL‐2024017).

## Conflicts of Interest

Min Chen and Kui Dong are employed by Shinall Technology. They are responsible for data collection and analysis. They have no other financial interests in the study and are unaware of the research design. Other authors disclose that there are no conflicts of interest to report.

## Supporting information




**TABLE S1**: Baseline characteristics of study population at tertiary hospital.
**TABLE S2**: Baseline characteristics of study population at non‐tertiary hospital.
**TABLE S3**: OAC prescription for overall, tertiary, and non‐tertiary participants.
**TABLE S4**: OAC prescription for individual tertiary and non‐tertiary hospitals.
**TABLE S5**: Different OAC drugs prescription for overall, tertiary, and non‐tertiary participants.
**TABLE S6**: OAC prescription for high‐risk and moderate stroke participants.
**TABLE S7**: Length of hospitalized stay and mortality.
**TABLE S8**: NOAC prices (¥) in different periods.

## Data Availability

The data underlying this article will be shared on reasonable request to the corresponding author.

## References

[1] G. Hindricks , T. Potpara , N. Dagres , et al., “2020 ESC Guidelines for the Diagnosis and Management of Atrial Fibrillation Developed in Collaboration With the European Association for Cardio‐Thoracic Surgery (EACTS): The Task Force for the Diagnosis and Management of Atrial Fibrillation of the European Society of Cardiology (ESC) Developed With the Special Contribution of the European Heart Rhythm Association (EHRA) of the ESC,” European Heart Journal 42, no. 5 (2021): 373–498. 32860505 10.1093/eurheartj/ehaa612

[2] J. A. Joglar , M. K. Chung , A. L. Armbruster , et al., “2023 ACC/AHA/ACCP/HRS Guideline for the Diagnosis and Management of Atrial Fibrillation: A Report of the American College of Cardiology/American Heart Association Joint Committee on Clinical Practice Guidelines,” Journal of the American College of Cardiology 149, no. 1 (2024):109‐279. 10.1161/CIR.0000000000001193 PMC11095842 38033089

[3] Y. Wang , Y. Guo , M. Qin , et al., “2024 Chinese Expert Consensus Guidelines on the Diagnosis and Treatment of Atrial Fibrillation in the Elderly, Endorsed by Geriatric Society of Chinese Medical Association (Cardiovascular Group) and Chinese Society of Geriatric Health Medicine (Cardiovascular Branch): Executive Summary,” Thrombosis and Haemostasis 124, no. 10 (2024): 897–911. 38744425 10.1055/a-2325-5923 PMC11436293

[4] I. C. Van Gelder , M. Rienstra , K. V. Bunting , et al., “2024 ESC Guidelines for the Management of Atrial Fibrillation Developed in Collaboration With the European Association for Cardio‐Thoracic Surgery (EACTS),” European Heart Journal 45, no. 36 (2024): 3314–3414. 39210723 10.1093/eurheartj/ehae176

[5] S. Shi , Y. Tang , Q. Zhao , et al., “Prevalence and Risk of Atrial Fibrillation in China: A National Cross‐Sectional Epidemiological Study,” The Lancet Regional Health Western Pacific 23 (2022): 100439. 35800039 10.1016/j.lanwpc.2022.100439 PMC9252928

[6] X. Liu , G. Feng , S. V. Marler , M. V. Huisman , G. Y. H. Lip , and C. Ma , “Real World Time Trends in Antithrombotic Treatment for Newly Diagnosed Atrial Fibrillation in China: Reports From the GLORIA‐AF Phase III Registry: Trends in Antithrombotic Therapy Use in China,” Thrombosis Journal 21, no. 1 (2023): 83. 37528405 10.1186/s12959-023-00527-x PMC10394786

[7] G. F. Romiti , B. Corica , M. Proietti , et al., “Patterns of Oral Anticoagulant Use and Outcomes in Asian Patients With Atrial Fibrillation: A Post‐Hoc Analysis From the GLORIA‐AF Registry,” EClinicalMedicine 63 (2023): 102039. 37753446 10.1016/j.eclinm.2023.102039 PMC10518516

[8] L. Shang , Y. Zhang , Y. Zhao , B. Tang , and Y. Hou , “Contemporary Oral Anticoagulant Therapy of Patients With Atrial Fibrillation in China: Status, Obstacles, and Strategies for Improvement,” Bioscience Trends 16, no. 5 (2022): 317–329. 36310085 10.5582/bst.2022.01278

[9] H. Q. Gu , X. Yang , C. J. Wang , et al., “Assessment of Trends in Guideline‐Based Oral Anticoagulant Prescription for Patients with Ischemic Stroke and Atrial Fibrillation in China,” JAMA Network Open 4, no. 7 (2021): e2118816. 34323982 10.1001/jamanetworkopen.2021.18816 PMC8322995

[10] M. Mazurek , M. V. Huisman , K. J. Rothman , et al., “Regional Differences in Antithrombotic Treatment for Atrial Fibrillation: Insights From the GLORIA‐AF Phase II Registry,” Thrombosis and Haemostasis 117, no. 12 (2017): 2376–2388. 29212125 10.1160/TH17-08-0555 PMC6260111

[11] Q. Y. Zhao , S. B. Shi , H. Huang , et al., “Contemporary Characteristics, Management, and Outcomes of Patients Hospitalized for Atrial Fibrillation in China: Results From the Real‐World Study of Chinese Atrial Fibrillation Registry,” Chinese Medical Journal 133, no. 23 (2020): 2883–2884. 33273341 10.1097/CM9.0000000000001151 PMC10631585

[12] J. Lin , D. Long , C. Jiang , et al., “Oral Anti‐Coagulants Use in Chinese Hospitalized Patients With Atrial Fibrillation,” Chinese Medical Journal 137, no. 2 (2024): 172–180. 38146256 10.1097/CM9.0000000000002915 PMC10798766

[13] X. Gong , Q. He , J. Yan , et al., “A Drug Utilization Study of Oral Anticoagulants in Five Representative Cities of China Between 2015 and 2019,” Journal of Clinical Pharmacy and Therapeutics 47, no. 1 (2022): 38–45. 34592785 10.1111/jcpt.13538

[14] H. Zhou , X. Nie , M. Jiang , and W. Dong , “Cost‐Effectiveness of Anticoagulants for Preventing Stroke in Patients With Non‐Valvular Atrial Fibrillation in Mainland China,” Journal of Clinical Pharmacy and Therapeutics 47, no. 4 (2022): 523–530. 34783090 10.1111/jcpt.13575

[15] J. Shen , Y. Xia , S. Cao , et al., “Knowledge, Attitude, and Practice Regarding Atrial Fibrillation Among Neurologists in Central China: A Cross‐Sectional Study,” Clinical Cardiology 43, no. 6 (2020): 639–646. 32208538 10.1002/clc.23361 PMC7298974

[16] K. A. Martinez , H. M. Hurwitz , and M. B. Rothberg , “Qualitative Analysis of Patient‒Physician Discussions Regarding Anticoagulation for Atrial Fibrillation,” JAMA Internal Medicine 182, no. 12 (2022): 1260–1266. 36315125 10.1001/jamainternmed.2022.4918 PMC9623476

[17] X. Du , L. Guo , S. Xia , et al., “Atrial Fibrillation Prevalence, Awareness and Management in a Nationwide Survey of Adults in China,” Heart 107, no. 7 (2021): 535–541. 33509976 10.1136/heartjnl-2020-317915 PMC7958113

[18] L. Beier , S. Lu , L. R. Franca , et al., “Evolution of Antithrombotic Therapy for Patients With Atrial Fibrillation: The Prospective Global GLORIA‐AF Registry Program,” PLoS One 17, no. 10 (2022): e0274237. 36201473 10.1371/journal.pone.0274237 PMC9536607

[19] S. Xue , X. Qiu , M. Wei , et al., “Changing Trends and Factors Influencing Anticoagulant Use in Patients With Acute Ischemic Stroke and NVAF at Discharge in the NOACs Era,” Journal of Stroke and Cerebrovascular Diseases 32, no. 2 (2023): 106905. 36473400 10.1016/j.jstrokecerebrovasdis.2022.106905

[20] B. A. Steinberg , H. Gao , P. Shrader , et al., “International Trends in Clinical Characteristics and Oral Anticoagulation Treatment for Patients With Atrial Fibrillation: Results From the GARFIELD‐AF, ORBIT‐AF I, and ORBIT‐AF II Registries,” American Heart Journal 194 (2017): 132–140. 29223431 10.1016/j.ahj.2017.08.011

[21] O. Y. Bang , Y. K. On , M. Y. Lee , et al., “The Risk of Stroke/Systemic Embolism and Major Bleeding in Asian Patients With Non‐Valvular Atrial Fibrillation Treated With Non‐Vitamin K Oral Anticoagulants Compared to Warfarin: Results From a Real‐World Data Analysis,” PLoS One 15, no. 11 (2020): e0242922. 33253294 10.1371/journal.pone.0242922 PMC7703907

[22] J. Oldgren , S. Asberg , Z. Hijazi , et al., “Early Versus Delayed Non‐Vitamin K Antagonist Oral Anticoagulant Therapy After Acute Ischemic Stroke in Atrial Fibrillation (TIMING): A Registry‐Based Randomized Controlled Noninferiority Study,” Circulation 146, no. 14 (2022): 1056–1066. 36065821 10.1161/CIRCULATIONAHA.122.060666 PMC9648987

[23] P. E. Papakonstantinou , V. Kalogera , D. Charitos , et al., “When Anticoagulation Management in Atrial Fibrillation Becomes Difficult: Focus on Chronic Kidney Disease, Coagulation Disorders, and Cancer,” Blood Reviews 65 (2024): 101171. 38310007 10.1016/j.blre.2024.101171

[24] L. Drikite , J. P. Bedford , L. O'Bryan , et al., “Treatment Strategies for New Onset Atrial Fibrillation in Patients Treated on an Intensive Care Unit: A Systematic Scoping Review,” Critical Care (London, England) 25, no. 1 (2021): 257. 34289899 10.1186/s13054-021-03684-5 PMC8296751

[25] M. K. Wang , R. Heo , P. Meyre , et al., “Use of Anticoagulation Therapy in Patients with Perioperative Atrial Fibrillation After Cardiac Surgery: A Systematic Review and Meta‐Analysis,” CJC Open 4, no. 10 (2022): 840–847. 36254332 10.1016/j.cjco.2022.06.003 PMC9568684

[26] Y. G. Li , S. R. Lee , E. K. Choi , and G. Y. Lip , “Stroke Prevention in Atrial Fibrillation: Focus on Asian Patients,” Korean Circulation Journal 48, no. 8 (2018): 665–684. 30073805 10.4070/kcj.2018.0190 PMC6072666

[27] C. Liu , X. Du , C. Jiang , et al., “Long‐Term Persistence With Newly‐Initiated Warfarin or Non‐VKA Oral Anticoagulant (NOAC) in Patients With Non‐Valvular Atrial Fibrillation: Insights From the Prospective China‐AF Registry,” Medical Science Monitor 25 (2019): 2649–2657. 30971681 10.12659/MSM.915875 PMC6475121

[28] J. J. Komen , A. Pottegard , A. K. Mantel‐Teeuwisse , et al., “Persistence and Adherence to Non‐Vitamin K Antagonist Oral Anticoagulant Treatment in Patients With Atrial Fibrillation Across Five Western European Countries,” Europace 23, no. 11 (2021): 1722–1730. 34096584 10.1093/europace/euab091 PMC8576279

[29] S. J. Zhao , B. Y. Chen , X. J. Hong , et al., “Prevalence, Risk Factors, and Prediction of Inappropriate Use of Non‐Vitamin K Antagonist Oral Anticoagulants in Elderly Chinese Patients With Atrial Fibrillation: A Study Protocol,” Frontiers in Cardiovascular Medicine 9 (2022): 951695. 36093129 10.3389/fcvm.2022.951695 PMC9449806

[30] Z. Ding , C. Zhang , Y. Y. Qian , et al., “Rationale and Design of a Prospective, Multicenter, Cross‐Sectional Study of Appropriateness Evaluation of the Prescription of Non‐Vitamin K Antagonist Oral Anticoagulants for Chinese Atrial Fibrillation Patients (Chi‐NOACs‐AF Trial),” Annals of Translational Medicine 9, no. 7 (2021): 580. 33987278 10.21037/atm-20-6893 PMC8105835

[31] T. Bucci , M. Proietti , A. Shantsila , et al., “Integrated Care for Atrial Fibrillation Using the ABC Pathway in the Prospective APHRS‐AF Registry,” JACC Asia 3, no. 4 (2023): 580–591. 37614548 10.1016/j.jacasi.2023.04.008 PMC10442886

[32] S. Shi , Q. Zhao , T. Liu , et al., “Left Atrial Thrombus in Patients with Non‐Valvular Atrial Fibrillation: A Cross‐Sectional Study in China,” Frontiers in Cardiovascular Medicine 9 (2022): 827101. 35586655 10.3389/fcvm.2022.827101 PMC9109812

