# Supplementary material for: Oral Anticoagulant Therapy of Patients With Atrial Fibrillation in Cardiology, Internal Medicine, and Surgery: Temporal Trend
Source: MedComm (2020). 2025 Nov 16;6(12):e70487. doi: 10.1002/mco2.70487 (PMC12620555; doi:10.1002/mco2.70487)
Supplement: Supplementary file 1 — TABLE S1: Baseline characteristics of study population at tertiary hospital. TABLE S2: Baseline characteristics of study population at non‐tertiary hospital. TABLE S3: OAC prescription for overall, tertiary, and non‐tertiary participants. TABLE S4: OAC prescription for individual tertiary and non‐tertiary hospitals. TABLE S5: Different OAC drugs prescription for overall, tertiary, and non‐tertiary participants. TABLE S6: OAC prescription for high‐risk and moderate stroke participants. TABLE S7: Length of hospitalized stay and mortality. TABLE S8: NOAC prices (¥) in different periods. [file MCO2-6-e70487-s001.docx]

Oral Anticoagulant Therapy of Patients with Atrial Fibrillation in Cardiology, Internal Medicine, and Surgery: Temporal Trend

Mingjie Lin, PhD^1,2,#^; He Huang, MD^3,#^; Juntao Wang, MD^1,2^; Hui Sun, MD^1^; Xingsheng Xu, MD^1^; Yan Zhang, MD^1^; Wenqiang Han, MD^2^; Min Chen, MS^4^; Kui Dong, MS^4^; Yingcui Wang, MD^1^; Beian You, ,MD^1^; Guihua Yao, MD^1^; Jingquan Zhong, PhD, MD^1,2,*^; Congxin Huang, MD^3,**^

1. Department of Cardiology, Qilu Hospital (Qingdao), Cheeloo College of Medicine, Shandong University, Qingdao, China
2. State Key Laboratory for Innovation and Transformation of Luobing Theory; Key Laboratory of Cardiovascular Remodeling and Function Research, Chinese Ministry of Education, Chinese National Health Commission and Chinese Academy of Medical Sciences; Department of Cardiology, Qilu Hospital of Shandong University, Jinan, China
3. Department of Cardiology, Renmin Hospital of Wuhan University, Wuhan, China
4. Shinall Technology, Wuhan, China

^#^ They made equal contributions to the study.

Running title: Anticoagulant Therapy Change in China

* Corresponding author. Department of Cardiology, Qilu Hospital of Shandong University, Jinan, China. [198762000778@email.sdu.edu.cn](mailto:198762000778@email.sdu.edu.cn), Phone: +86 18560086597.

** Corresponding author. Department of Cardiology, Renmin Hospital of Wuhan University, Wuhan, China. [huangcongxin@vip.163.com](mailto:huangcongxin@vip.163.com).


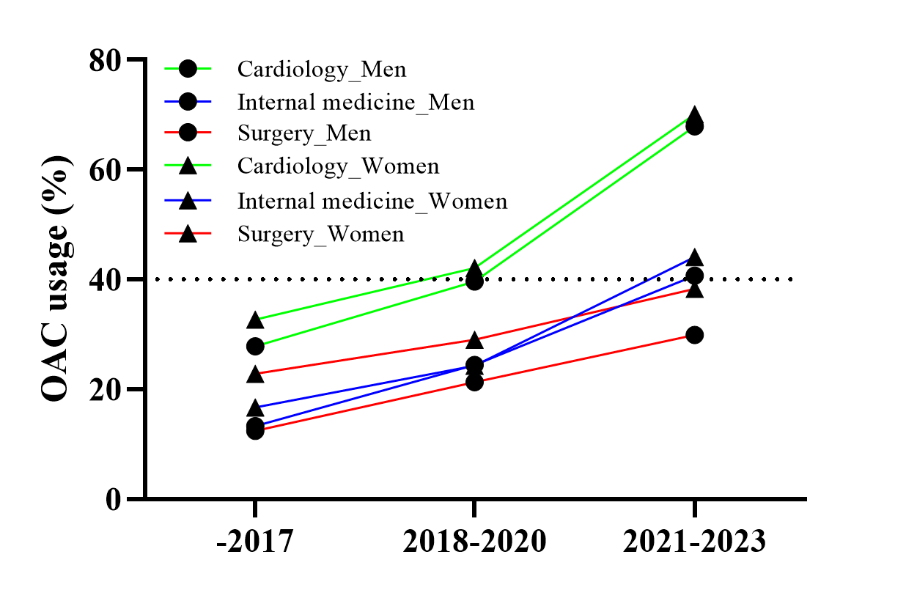


Supplementary Figure S1. Temporal trend of anticoagulant prescription in sex-based patients with AF.

| Supplementary Table S1. Baseline characteristics of study population at tertiary hospital | | | | |
| --- | --- | --- | --- | --- |
|  | Overall (n=24278) | Cardiology (n=9146) | Internal Medicine (n=11065) | Surgery (n=4067) |
| Age (years) |  |  |  |  |
| 65-74 | 7549(31.09) | 3207(35.06) | 3114(28.14)& | 1228(30.19)& |
| ≧75 | 10984(45.24) | 3520(38.49) | 5722(51.71)& | 1742(42.83)& |
| Male | 14129(58.19) | 5195(56.8) | 6496(58.71)# | 2438(59.95)& |
| Congestive heart failure | 5263(21.68) | 2355(25.75) | 2502(22.61)& | 406(9.98)& |
| Hypertension | 13352(55.0) | 5049(55.2) | 6295(56.89)* | 2008(49.37)& |
| Diabetes mellitus | 5273(21.72) | 1901(20.79) | 2657(24.01)& | 715(17.58)& |
| Stroke/TIA/systemic embolism | 5808(23.92) | 1364(14.91) | 3623(32.74)& | 821(20.19)& |
| Vascular disease | 2924(12.04) | 1139(12.45) | 1528(13.81)# | 257(6.32)& |
| CHA2DS2-VASc Score |  |  |  |  |
| 0 | 1197(4.93) | 550(6.01) | 365(3.30) | 282(6.93) |
| 1 | 3012(12.41) | 1282(14.02) | 1063(9.61) | 667(16.40) |
| 2 | 4568(18.82) | 1848(20.21) | 1807(16.33) | 913(22.45) |
| 3 | 5295(21.81) | 2074(22.68) | 2303(20.81) | 918(22.57) |
| 4 | 4571(18.83) | 1680(18.37) | 2230(20.15) | 661(16.25) |
| 5 | 3051(12.57) | 1036(11.33) | 1647(14.88) | 368(9.05) |
| 6 | 1730(7.13) | 493(5.39) | 1056(9.54) | 181(4.45) |
| 7 | 699(2.88) | 153(1.67) | 481(4.35) | 65(1.60) |
| 8 | 146(0.60) | 29(0.32) | 106(0.96) | 11(0.27) |
| 9 | 9(0.04) | 1(0.01) | 7(0.06) | 1(0.02) |
| Renal disease | 2801(11.54) | 1118(12.22) | 1404(12.69) | 279(6.86)& |
| Liver disease | 1304(5.37) | 464(5.07) | 619(5.59) | 221(5.43) |
| Anemia | 1998(8.23) | 601(6.57) | 1075(9.72)& | 322(7.92)# |
| Bleeding history |  |  |  |  |
| Cerebral hemorrhage | 369(1.52) | 67(0.73) | 202(1.83)& | 100(2.46)& |
| Gastrointestinal bleeding | 647(2.66) | 100(1.09) | 472(4.27)& | 75(1.84)# |
| Other bleeding diseases | 506(2.08) | 45(0.49) | 261(2.36)& | 200(4.92)& |
| Hyperthyroidism | 490(2.02) | 206(2.25) | 233(2.11) | 51(1.25)& |
| Malignant tumor | 1690(6.96) | 117(1.28) | 733(6.62)& | 840(20.65)& |
| Hemoglobin level (g/l) |  |  |  |  |
| 90-120 | 2012(8.29) | 694(7.59) | 940(8.5)* | 378(9.29)# |
| 60-90 | 456(1.88) | 115(1.26) | 248(2.24)& | 93(2.29)& |
| <60 | 63(0.26) | 8(0.09) | 45(0.41)& | 10(0.25)* |
| Platelet (10^9/l) |  |  |  |  |
| 50-100 | 1034(4.26) | 374(4.09) | 480(4.34) | 180(4.43) |
| <50 | 251(1.03) | 49(0.54) | 167(1.51)& | 35(0.86)* |
| OAC | 13437(55.35) | 6537(71.47) | 5416(48.95)& | 1484(36.49)& |
| NOAC | 11437(47.11) | 5722(62.56) | 4670(42.21)& | 1045(25.69)& |
| Warfarin | 2371(9.77) | 994(10.87) | 894(8.08)& | 483(11.88) |
| Antiplatelet drugs | 8327(34.3) | 3857(42.17) | 3994(36.1)& | 476(11.7)& |
| Class I/III antiarrhythmic drugs | 4436(18.27) | 1977(21.62) | 1645(14.87)& | 814(20.01)* |
| β blockers | 13508(55.64) | 5761(62.99) | 5840(52.78)& | 1907(46.89)& |
| Chinese medicine | 1427(5.88) | 771(8.43) | 520(4.70)& | 136(3.34)& |
| Other antiarrhythmic drugs | 6877(28.33) | 3070(33.57) | 2522(22.79)& | 1285(31.6)* |
| ACEI/ARB | 13658(56.26) | 6400(69.98) | 6256(56.54)& | 1002(24.64)& |
| Statins | 1668(6.87) | 1065(11.64) | 504(4.55)& | 99(2.43)& |
| SGLT2i | 10189(41.97) | 4715(51.55) | 4350(39.31)& | 1124(27.64)& |
| MRAs | 9245(38.08) | 4578(50.05) | 3762(34.0)& | 905(22.25)& |
| vs. cardiology *p<0.05, #p<0.01, &p<0.001 | | | | |
| ALT, alanine transaminase; AST, aspartate aminotransferase; OAC, oral anticoagulants; NOAC, non-VKA oral anticoagulants; ACEI, angiotensin converting enzyme inhibitors; ARB, angiotensin receptor inhibitor; SGLT2i, sodium glucose cotransporter inhibitors; MRAs, mineralocorticoid receptor antagonists | | | | |

| Supplementary Table S2. Baseline characteristics of study population at nontertiary hospital | | | | |
| --- | --- | --- | --- | --- |
|  | Overall (n=45909) | Cardiology (n=17994) | Internal Medicine (n=23760) | Surgery (n=4155) |
| Age (years) |  |  |  |  |
| 65-74 | 14817(32.27%) | 5990(33.29%) | 7447(31.34%)& | 1380(33.21%) |
| ≧75 | 21416(46.65) | 7611(42.3%) | 12114(50.98%)& | 1691(40.7%) |
| Male | 24065(52.41) | 9104(50.59) | 12644(53.22)& | 2317(55.76)& |
| Congestive heart failure | 7920(17.25) | 4178(23.22) | 3458(14.55)& | 284(6.84)& |
| Hypertension | 22329(48.64) | 9023(50.14) | 11638(48.98)* | 1668(40.14)& |
| Diabetes mellitus | 7875(17.15) | 3193(17.74) | 4123(17.35) | 559(13.45)& |
| Stroke/TIA/systemic embolism | 12434(27.08) | 2636(14.65) | 8961(37.71)& | 837(20.14)& |
| Vascular disease | 7082(15.43) | 2659(14.78) | 4124(17.36)& | 299(7.2)& |
| CHA2DS2-VASc Score |  |  |  |  |
| 0 | 2023(4.41) | 962(5.35) | 774(3.26) | 287(6.91) |
| 1 | 5392(11.74) | 2428(13.49) | 2194(9.23) | 770(18.53) |
| 2 | 8715(18.98) | 3726(20.71) | 3994(16.81) | 995(23.95) |
| 3 | 9898(21.56) | 4090(22.73) | 4891(20.59) | 917(22.07) |
| 4 | 8899(19.38) | 3442(19.13) | 4833(20.34) | 624(15.02) |
| 5 | 6113(13.32) | 2026(11.26) | 3743(15.75) | 344(8.28) |
| 6 | 3396(7.4) | 927(5.15) | 2298(9.67) | 171(4.12) |
| 7 | 1279(2.79) | 332(1.85) | 902(3.8) | 45(1.08) |
| 8 | 185(0.4) | 56(0.31) | 127(0.53) | 2(0.05) |
| 9 | 9(0.02) | 5(0.03) | 4(0.02) | 0(0.0) |
| Renal disease | 2699(5.88) | 1149(6.39) | 1392(5.86)* | 158(3.8)& |
| Liver disease | 1297(2.83) | 477(2.65) | 674(2.84) | 146(3.51)# |
| Anemia | 3095(6.74) | 944(5.25) | 1830(7.7)& | 321(7.73)& |
| Bleeding history |  |  |  |  |
| Cerebral hemorrhage | 598(1.3) | 104(0.58) | 319(1.34)& | 175(4.21)& |
| Gastrointestinal bleeding | 1202(2.62) | 220(1.22) | 864(3.64)& | 118(2.84)& |
| Other bleeding diseases | 787(1.71) | 43(0.24) | 415(1.75)& | 329(7.92)& |
| Hyperthyroidism | 929(2.02) | 421(2.34) | 469(1.97)* | 39(0.94)& |
| Malignant tumor | 1426(3.11) | 150(0.83) | 687(2.89)& | 589(14.18)& |
| Hemoglobin level (g/l) |  |  |  |  |
| 90-120 | 2531(5.51) | 1071(5.95) | 1238(5.21) | 222(5.34) |
| 60-90 | 646(1.41) | 292(1.62) | 277(1.17) | 77(1.85) |
| <60 | 115(0.25) | 53(0.29) | 55(0.23) | 7(0.17) |
| Platelet (10^9/l) |  |  |  |  |
| 50-100 | 9(0.02) | 2(0.01) | 4(0.02) | 3(0.07) |
| <50 | 3(0.01) | 0(0.0) | 3(0.01) | 0(0.0) |
| OAC | 16716(36.41) | 9524(52.93) | 6214(26.15)& | 978(23.54)& |
| NOAC | 11135(24.25) | 6675(37.1) | 3962(16.68)& | 498(11.99)& |
| Warfarin | 5944(12.95) | 3092(17.18) | 2350(9.89)& | 502(12.08)& |
| Antiplatelet drugs | 20113(43.81) | 8104(45.04) | 11413(48.03)& | 596(14.34)& |
| Class I/III antiarrhythmic drugs | 7401(16.12) | 3693(20.52) | 3021(12.71)& | 687(16.53)& |
| β blockers | 22567(49.16) | 11216(62.33) | 9805(41.27)& | 1546(37.21)& |
| Chinese medicine | 3096(6.74) | 1635(9.09) | 1369(5.76)& | 92(2.21)& |
| Other antiarrhythmic drugs | 6731(14.66) | 3018(16.77) | 2761(11.62)& | 952(22.91)& |
| ACEI/ARB | 28183(61.39) | 12715(70.66) | 14492(60.99)& | 976(23.49)& |
| Statins | 2479(5.4) | 1872(10.4) | 530(2.23)& | 77(1.85)& |
| SGLT2i | 17020(37.07) | 9543(53.03) | 6665(28.05)& | 812(19.54)& |
| MRAs | 19036(41.46) | 9714(53.98) | 8526(35.88)& | 796(19.16)& |
| vs. cardiology *p<0.05, #p<0.01, &p<0.001 | | | | |
| ALT, alanine transaminase; AST, aspartate aminotransferase; OAC, oral anticoagulants; NOAC, non-VKA oral anticoagulants; ACEI, angiotensin converting enzyme inhibitors; ARB, angiotensin receptor inhibitor; SGLT2i, sodium glucose cotransporter inhibitors; MRAs, mineralocorticoid receptor antagonists | | | | |

| Supplementary Table S3. OAC prescription for overall, tertiary, nontertiery participants | | | |
| --- | --- | --- | --- |
| Overall | Cardiology | Internal medicine | Surgery |
| -2017 | 545（29.8%） | 651（14.8%）* | 104（17.3%）* |
| 2018-2020 | 2869（41.2%） | 2597（24.4%）* | 527（24.6%）* |
| 2021-2023 | 12578（68.8%） | 8300（42.2%）* | 1823（33.4%）* |
| Tertiery | Cardiolgoy | Internal medicine | Surgery |
| -2017 | 0 | 0 | 0 |
| 2018-2020 | 577 (43.48%) | 1004 (51.62%)* | 255 (39.05%)* |
| 2021-2023 | 5955 (76.21%) | 4411 (48.37%)* | 1229 (36.0%)* |
| Nontertiery | Cardiolgoy | Internal medicine | Surgery |
| -2017 | 545 (29.86) | 651 (14.89%)* | 104 (17.28%)* |
| 2018-2020 | 2292（40.62%） | 1593 (18.33%)* | 272 (18.28)* |
| 2021-2023 | 6623 (63.38%) | 3889 (36.94%)* | 594 (29.05%)* |
|  |  |  |  |
| *p<0.05 vs. cardiology |  |  |  |

| Supplementary Table S4. OAC prescription for individual tertiary and nontertiery hospitals | | | | | |
| --- | --- | --- | --- | --- | --- |
| Hospital | | Overall | -2017 | 2018-2020 | 2021-2023 |
| Teriary | No.1 | 2248(58.7%) |  |  | 2248(58.7%) |
|  | No.2 | 982(48.9%) |  | 316(33.7%) | 666(62.2%) |
|  | No.3 | 783(37.8%) |  |  | 783(37.8%) |
|  | No.4 | 4211(56.5%) |  | 634(49.9%) | 3577(57.8%) |
|  | No.5 | 2022(51.5%) |  | 862(52.1%) | 1160(51.0%) |
|  | No.6 | 3191(64.1%) |  |  | 3191(64.1%) |
| Nonteritiary | No.1 | 568(15.5%) | 64(6.6%) | 111(6.7%) | 393(38.9%) |
|  | No.2 | 598(19.3%) | 138(10.9%) | 344(24.1%) | 116(28.2%) |
|  | No.3 | 529(81.5%) |  | 373(79.7%) | 156(86.2%) |
|  | No.4 | 19(70.4%) |  |  | 19(70.4%) |
|  | No.5 | 910(37.7%) | 217(28.8%) | 258(34.9%) | 435(47.3%) |
|  | No.6 | 4749(51.6%) | 128(23.7%) | 1438(43.9%) | 3183(59.1%) |
|  | No.7 | 741(60.0%) |  |  | 741(60.0%) |
|  | No.8 | 1190(62.7%) |  |  | 1190(62.7%) |
|  | No.9 | 245(19.5%) | 20(7.3%) | 27(5.5%) | 198(40.5%) |
|  | No.10 | 443(31.9%) |  | 0(0%) | 443(32.0%) |
|  | No.11 | 2681(29.9%) | 704(25.2%) | 1020(29.8%) | 957(32.6%) |
|  | No.12 | 855(61.1%) |  |  | 855(61.1%) |
|  | No.13 | 1393(16.6%) | 1(8.3%) | 487(11.1%) | 905(22.6%) |
|  | No.14 | 1795(77.9%) | 3(9.7%) | 78(56.1%) | 1714(80.3%) |

| Supplementary Table S5. Different OAC drugs prescription for overall, tertiary, nontertiery participants | | | | | | | | | |
| --- | --- | --- | --- | --- | --- | --- | --- | --- | --- |
|  | Warfarin | | | Dabigatran | | | Rivaroxaban | | |
| Overall | Cardiology | Internal medicine | Surgery | Cardiology | Internal medicine | Surgery | Cardiology | Internal medicine | Surgery |
| -2017 | 221 (40.55%) | 410 (62.98%) | 21 (20.19%) | 84 (15.41%) | 12 (1.84%) | 0 | 0 | 0 | 4 (3.85%) |
| 2018-2020 | 1098 (38.27%) | 1015 (39.08%) | 102 (19.35%) | 592 (20.63%) | 343 (13.21%) | 54 (10.25%) | 643 (22.41%) | 941 (36.23%) | 178 (33.78%) |
| 2021-2023 | 1050 (8.35%) | 733 (8.83%) | 160 (8.78%) | 742 (5.9%) | 693 (8.35%) | 72 (3.95%) | 10393 (82.63%) | 6601 (79.53%) | 1199 (65.77%) |
|  |  | |  |  |  |  |  |  |  |
|  | Warfarin | | | Dabigatran | | | Rivaroxaban | | |
| Tertiery | Cardiology | Internal medicine | Surgery | Cardiology | Internal medicine | Surgery | Cardiology | Internal medicine | Surgery |
| -2017 | 0 | 0 | 0 | 0 | 0 | 0 | 0 | 0 | 0 |
| 2018-2020 | 169 (29.29%) | 166 (16.53%) | 28 (10.98%) | 156 (27.04%) | 154 (15.34%) | 41 (16.08%) | 225 (38.99%) | 636 (63.35%) | 113 (44.31%) |
| 2021-2023 | 345 (5.79%) | 350 (7.93%) | 76 (6.18%) | 430 (7.22%) | 462 (10.47%) | 58 (4.72%) | 4989 (83.78%) | 3450 (78.21%) | 833 (67.78%) |
|  |  |  |  |  |  |  |  |  |  |
|  | Warfarin | | | Dabigatran | | | Rivaroxaban | | |
| Nontertiery | Cardiology | Internal medicine | Surgery | Cardiology | Internal medicine | Surgery | Cardiology | Internal medicine | Surgery |
| -2017 | 221 (40.55%) | 410 (62.98%) | 21 (20.19%) | 84 (15.41%) | 12 (1.84%) | 0 | 0 | 0 | 4 (3.85%) |
| 2018-2020 | 929 (40.53%) | 849 (53.3%) | 74 (27.21%) | 436 (19.02%) | 189 (11.86%) | 13 (4.78%) | 418 (18.24%) | 305 (19.15%) | 65 (23.9%) |
| 2021-2023 | 705 (10.64%) | 383 (9.85%) | 84(14.14%) | 312 (4.71%) | 231 (5.94%) | 14 (2.36%) | 5404 (81.59%) | 3151 (81.02%) | 366 (61.62%) |

| Supplementary Table S6. OAC prescription for high-risk and moderate stroke participants | | | |
| --- | --- | --- | --- |
| High-risk stroke patients | OAC | | |
|  | Cardiology | Internal medicine | Surgery |
| -2017 | 322 (27.15%） | 431 (13.17%) | 27 (7.65%) |
| 2018-2020 | 1927（39.56%） | 2064 (23.51%) | 291 (20.45%) |
| 2021-2023 | 9466 （68.29%） | 7239 (43.24%) | 1238 (32.11%) |
|  |  |  |  |
| Moderate-risk stroke patients | OAC | | |
|  | Cardiology | Internal medicine | Surgery |
| -2017 | 129 (32.0%) | 137 (19.8%) | 26 (20.3%) |
| 2018-2020 | 580 (44.1%) | 356 (29.2%) | 90 (22.9% |
| 2021-2023 | 2092 (72.0%) | 785 (38.1%) | 355 (35.1%) |

| Supplementary Table S7. Length of hospitalized stay and mortality | | | |
| --- | --- | --- | --- |
| Hospitalized stay | Cardiology | Internal medicine | Surgery |
| OAC therapy (day) | 7.39(4.45) | 9.11(7.03) | 13.79(10.17) |
| Non-OAC therapy（day） | 6.90(4.79)* | 8.63(7.41)* | 10.36(9.13)* |
| Mortality |  |  |  |
| OAC therapy (N,‰) | 3(0.19) | 2(0.17) | 0 |
| Non-OAC therapy（N,‰） | 7(0.63) | 57(2.46)* | 6(1.04) |

| Supplementary Table S8. NOAC prices (¥) in different periods | | | |  |
| --- | --- | --- | --- | --- |
| Types of NOAC | Prior 2018* | 2018 to 2020 | 2021 to 2023 | since 2023 |
| Rivaroxaban (20mg*7 pieces) | 450 | 280 | 150 | 130 |
| Generic Rivaroxaban (20mg*28 pieces) | - | - | - | 30-50 |
| Dabigatran (110mg*10 pieces) | 500 | 320 | 180 | 150 |
| Generic Dabigatran (110mg*28 pieces) | - | - | - | 50 |
| Note: Depending on the type of insurance, patients pay 10-50% out of pocket | | | | |
| * Prior 2018, patients have to pay the full cost | | | | |
